# The Association Between Serum C-Reactive Protein Levels and Body Fat Parameters: Results from the Korean National Health and Nutrition Examination Survey

**DOI:** 10.3390/medicina62061014

**Published:** 2026-05-23

**Authors:** Hyemin Jeong

**Affiliations:** Division of Rheumatology, Department of Internal Medicine, Soonchunhyang University Bucheon Hospital, Bucheon 14584, Republic of Korea; hm.jeong@schmc.ac.kr; Tel.: +82-32-621-5017

**Keywords:** C-reactive protein, fat mass index, trunk fat, KNHANES

## Abstract

*Background and Objectives*: C-reactive protein (CRP), a marker of systemic inflammation, is frequently elevated in individuals with excessive adiposity. However, the relationship between specific body fat parameters and CRP levels across sexes and age groups remains unclear. This study aimed to investigate the association between CRP levels and body fat parameters using data from the 2022 Korea National Health and Nutrition Examination Survey. *Methods*: A total of 3369 participants (representing 32,635,626 Korean adults) were included. Demographic, lifestyle, and clinical data were collected, and body composition parameters were measured using bioelectrical impedance analysis. The primary exposure variables included fat mass index (FMI; total body fat mass/height^2^) and trunk fat mass. The primary outcome was log-transformed high-sensitivity CRP (ln[hsCRP]). Pearson correlation and sex-stratified multivariate linear regression analyses were performed. *Results*: The mean age of participants was 49.6 years, and 53.3% were male. ln[hsCRP] was positively associated with body mass index, waist circumference, total body fat mass, FMI, appendicular fat mass, and trunk fat mass in both sexes (all *p* < 0.001). FMI showed a stronger association with ln[hsCRP] in females (r = 0.373) than in males (r = 0.232). In multivariable analyses, FMI remained independently associated with ln[hsCRP] in both males (β = 0.10) and females (β = 0.14), with a stronger effect observed in females. Trunk fat mass was also independently associated with ln[hsCRP] (β = 0.06 in males; β = 0.10 in females). Age-stratified analyses demonstrated that these associations were more pronounced in younger adults (19–40 years old) than in those aged 41–70 years. *Conclusions*: In Korean adults, total and truncal fat masses were independently associated with systemic inflammation, and this association was stronger among females and younger adults.

## 1. Introduction

Low-grade systemic inflammation is a critical component of the pathogenesis of various chronic diseases, including cardiovascular diseases, diabetes, and metabolic syndrome [1,2]. C-reactive protein (CRP), particularly high-sensitivity CRP (hsCRP), is a well-recognized and routinely measured inflammation biomarker. Elevated CRP levels are strongly associated with an increased risk of morbidity and mortality associated with cardiometabolic disorders [3]. Obesity is a major contributor to chronic inflammation [4]. Adipose tissue, traditionally regarded as merely an energy storage organ, is now considered an active endocrine organ that secretes numerous pro-inflammatory cytokines or adipokines, such as tumor necrosis factor-alpha (TNF-α) and interleukin-6 (IL-6) [5]. These cytokines stimulate the liver to synthesize acute-phase proteins, including CRP, thereby establishing a direct link between excess adiposity and systemic inflammation [6].

Body mass index (BMI) has conventionally been used to assess obesity. However, BMI is an inadequate measure of adiposity, as it does not differentiate between lean muscle mass and fat mass or account for fat distribution. Body composition analysis, often conducted using bioelectrical impedance analysis (BIA), provides more detailed and accurate measures of adiposity, such as total body fat mass (BFM), percentage body fat (PBF), and regional fat deposits, including trunk fat mass. Furthermore, fat mass index (FMI), calculated as BFM divided by height squared, is considered a more specific measure of adiposity than BMI [7].

Body fat distribution is an important determinant of metabolic health. Trunk fat mass, representing visceral and subcutaneous fat accumulation in the abdominal area, is particularly important in driving metabolic dysfunction and inflammation [8]. Previous studies have indicated that central adiposity is a stronger risk factor for chronic diseases than overall obesity [9].

Furthermore, sex- and age-specific differences in fat distribution may alter the association between adiposity and inflammation. Females tend to have higher overall body fat percentages, whereas males tend to accumulate more visceral fat, which may lead to different inflammatory responses [10]. Previous studies using data from the Korea National Health and Nutrition Examination Survey (KNHANES) have demonstrated associations between CRP concentrations and the increased prevalence of hypertriglyceridemia and metabolic syndrome [11,12]. However, these studies have primarily relied on BMI or waist circumference when evaluating the relationship between CRP and metabolic conditions. Importantly, BIA was newly introduced in the 2022 KNHANES, enabling the nationwide assessment of detailed body composition parameters. Studies analyzing the relationship between hsCRP and detailed adiposity markers in large representative populations, particularly Asian populations, remain limited. The KNHANES provides high-quality nationwide data on detailed body composition parameters and hsCRP levels.

The prevalence of obesity and adiposity-related metabolic disorders has increased globally, including in South Korea, with a notable upward trend despite lower overall rates than those in Western populations. Moreover, Asian populations may have higher body fat and metabolic risk at lower BMI levels [13,14]. These observations underscore the importance of a detailed assessment of body fat parameters and their relationship with systemic inflammation. This study aimed to investigate the association between hsCRP levels and body fat parameters using data from the 2022 KNHANES and assess whether these associations differed by sex and age group.

## 2. Materials and Methods

### 2.1. Study Population

Data from the 2022 KNHANES were analyzed. KNHANES is a nationwide periodic survey conducted by the Korea Disease Control and Prevention Agency to assess the health and nutritional status of the Korean population. By adopting a multistage stratified cluster sampling design with proportional allocation, the survey incorporated sample weights to generate estimates representative of the entire population. Among the 6265 participants in the 2022 KNHANES, 943 aged <19 years were excluded. Of the remaining 5322 adults, 1953 with missing values for independent or outcome variables were excluded. A total of 3369 participants (weighted *n* = 32,635,626) were included in the analysis.

This study was approved by the Institutional Review Board of the Soonchunhyang University Hospital (IRB No. 2026-04-021).

### 2.2. Data Collection

Demographic and socioeconomic data were collected using structured questionnaires during individualized interviews conducted by trained personnel. The participants provided information regarding their age, sex, smoking habits, alcohol intake, BMI, and physical activity levels. Regular alcohol consumption was defined as alcohol consumption at least once per month in the year preceding the survey. Aerobic exercise was categorized as achieving a minimum of 150 min of moderate-intensity activity per week, 75 min of vigorous-intensity activity, or an equivalent combination (where 1 min of vigorous activity corresponded to 2 min of moderate activity). Comorbidities, such as hypertension, dyslipidemia, and diabetes mellitus, were identified through physician-diagnosed conditions reported during standardized interviews. Participants were defined as having these conditions if they provided an affirmative response to the specific question, “Has a physician ever diagnosed you with [condition]?” (e.g., hypertension, dyslipidemia, or diabetes). Only those who responded “yes” were categorized as having the disorder. Blood pressure was measured in the right arm after a 5-min seated rest period using a mercury sphygmomanometer, and the mean of the final two of the three measurements was used for analysis. Following an 8-h overnight fast, blood samples were collected, immediately refrigerated, and transferred to a central laboratory facility under controlled cold-chain conditions.

### 2.3. Measurement of Body Composition

Body composition parameters were evaluated in 2022 using a multifrequency BIA system (InBody 970; InBody, Seoul, Korea). The device measured a range of parameters, including total BFM (kg), body fat percentage (%), lean body mass, muscle mass (excluding bone minerals), total body water, and whole-body phase angle. Participants with implantable pacemakers or cardioverter-defibrillators, as well as pregnant women, were excluded. Regional assessments were performed on the trunk and four extremities (right arm, left arm, right leg, and left leg). Appendicular fat mass (AFM) was calculated as the sum of fat mass from both arms and legs. To standardize body size, FMI was calculated by dividing total BFM (kg) by height squared (m^2^).

### 2.4. Statistical Analysis

Weighted analyses were performed to account for the complex, multistage, stratified, and clustered sampling design of KNHANES. Because the distribution of plasma CRP was highly skewed, log-transformed plasma CRP (ln[hsCRP]) was used for the analysis. No additional exclusion based on hsCRP thresholds was applied, as log transformation adequately mitigated the influence of extreme values. Continuous variables are presented as means ± standard errors (SEs), and categorical variables are reported as weighted percentages. Group comparisons were performed using weighted *t*-tests and chi-square (χ^2^) tests.

Multivariable linear regression models were constructed to evaluate the associations between fat mass and the outcome, with results presented as β coefficients and 95% confidence intervals (CIs). Two sequential adjustment models were used. Model 1 was adjusted for age, sex, smoking status, alcohol intake, and aerobic physical activity. Model 2 was further adjusted for comorbidities, including hypertension, diabetes, and dyslipidemia. Subgroup analyses stratified by sex and age were also performed, and the resulting *p*-values were adjusted using the Bonferroni method to account for multiple testing. Weighted Pearson correlation and survey-weighted regression analyses were performed. All statistical tests were two-sided, with a *p*-value < 0.05 considered statistically significant. Data were analyzed using SAS version 9.4 (SAS Institute Inc., Cary, NC, USA).

## 3. Results

### 3.1. Baseline Characteristics of the Study Population

A total of 3369 participants from the 2022 KNHANES were included in the final analysis, representing an estimated 32,635,626 individuals after applying sampling weights. Table 1 summarizes the demographic and clinical characteristics of the study population, categorized by sex. The mean age was 43.4 ± 0.6 years for males and 51.0 ± 0.6 years for females. The prevalence of current smoking was significantly higher among men (30.2%) than among women (4.4%), and alcohol consumption was more frequent among men than women (67.0% vs. 40.5%). The prevalence of hypertension and diabetes was higher in men than in women, whereas dyslipidemia was more prevalent in women than in men (25.4% vs. 19.1%). Regarding the body composition parameters, distinct sexual dimorphism was observed across several indices. While waist circumference (90.0 ± 0.3 cm vs. 81.2 ± 0.3 cm) and BMI (25.3 ± 0.1 vs. 24.0 ± 0.1 kg/m^2^) were higher in men than in women, adiposity-related parameters showed the opposite trend. PBF and FMI were markedly higher in women (34.04 ± 0.19% and 8.34 ± 0.09 kg/m^2^, respectively) than in men (25.42 ± 0.17% and 6.59 ± 0.07 kg/m^2^). AFM was also higher in females (9.26 ± 0.1 kg vs. 7.98 ± 0.09 kg in males). In contrast, trunk fat mass was comparable between the two groups. The mean hsCRP level was 1.38 ± 0.07 mg/L in the total population, with slightly higher values observed in males than in females.

### 3.2. Correlation Between ln[hsCRP] and Metabolic Parameters

Pearson correlation analysis revealed significant associations between ln[hsCRP] and several anthropometric and metabolic variables (Table 2). Body fat-related parameters showed the strongest correlation with ln[hsCRP]. In men, ln[hsCRP] was positively correlated with BFM (r = 0.218), PBF (r = 0.238), FMI (r = 0.232), AFM (r = 0.219), and trunk fat mass (r = 0.215; all *p* < 0.001). Similar but stronger correlations were observed in women, including BFM (r = 0.374), PBF (r = 0.330), FMI (r = 0.373), AFM (r = 0.373), and trunk fat mass (r = 0.367; all *p* < 0.001). Waist circumference, BMI, systolic blood pressure, and hemoglobin A1c, fasting glucose, and triglyceride levels were also positively correlated with ln[hsCRP] in both sexes. Conversely, high-density lipoprotein cholesterol was negatively correlated with ln[hsCRP] (men: r = −0.190; women: r = −0.228; both *p* < 0.001). Overall, the correlation between adiposity measurements and ln[hsCRP] levels was consistently stronger in women than in men.

### 3.3. Association Between FMI and Ln[hsCRP]

Linear regression analysis revealed a significant association between FMI and ln[hsCRP] (Table 3). In men, FMI was significantly associated with ln[hsCRP] in both univariate and multivariate models. After adjusting for age, smoking, alcohol consumption, and aerobic exercise (Model 1), the association remained significant (β = 0.10, 95% CI 0.08–0.12, *p* < 0.001). The association persisted after further adjustment for hypertension, dyslipidemia, and diabetes (Model 2) (β = 0.10, 95% CI, 0.08–0.12, *p* < 0.001). Similarly, in women, FMI was strongly associated with ln[hsCRP] in both the unadjusted and adjusted analyses. In the fully adjusted model (Model 2), the association remained significant (β = 0.14, 95% CI, 0.12–0.16, *p* < 0.001), with a larger effect size than that observed in men.

### 3.4. Association Between Trunk Fat Mass and ln[hsCRP]

Trunk fat mass was significantly associated with ln[hsCRP] in both sexes (Table 4). Among men, trunk fat mass showed a significant association with ln[hsCRP] in the fully adjusted model (β = 0.06, 95% CI 0.05–0.07, *p* < 0.001). Among women, the association was stronger, with a β coefficient of 0.10 (95% CI 0.09–0.12, *p* < 0.001) in the fully adjusted model. These findings suggest that truncal fat mass is independently associated with systemic inflammation, and that the magnitude of this association is greater in women than in men.

### 3.5. Association Between Adiposity and ln[hsCRP] According to Sex and Age

Table 5 shows the associations among FMI, trunk fat mass, and ln[hsCRP] stratified by sex and age. Both FMI and trunk fat mass were positively associated with ln[hsCRP] across all groups (all *p* < 0.001). Notably, these associations were more pronounced in younger adults (19–40 years) than in older adults (41–70 years). Among men, FMI showed a stronger association with ln[hsCRP] in younger individuals (β = 0.13) compared with older individuals (β = 0.09 in fully adjusted models). Similarly, trunk fat mass demonstrated a greater effect in younger men (β = 0.08) than in older men (β = 0.05). A similar pattern was observed in women, with a stronger overall association. In fully adjusted models, FMI was more strongly associated with ln[hsCRP] in younger women (β = 0.18) than in older women (β = 0.14). In addition, trunk fat mass showed a greater association in younger women (β = 0.14) than in older women (β = 0.10). Collectively, the associations between body fat parameters and systemic inflammation were consistently stronger in younger adults, with the most pronounced effects observed in younger women.

In interaction analyses, the associations of FMI and truncal fat mass with hsCRP significantly differed according to sex and age group (all interaction *p*-values < 0.01), with stronger associations observed in females and younger adults (Supplementary Table S1).

### 3.6. Association Between Adiposity Parameters and ln[hsCRP]

**Figure 1:**
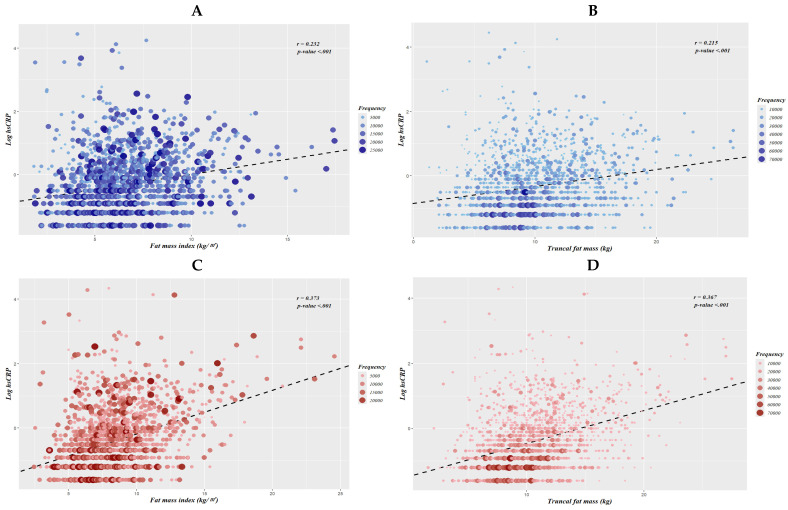
Association between adiposity measures and log-transformed hsCRP according to sex. Scatter plots showing the relationship between adiposity measures and log-transformed high-sensitivity C-reactive protein (ln[hsCRP]). (**A**) Association between fat mass index (FMI) and ln[hsCRP] in men. (**B**) Association between truncal fat mass and ln[hsCRP] in men. (**C**) Association between FMI and ln[hsCRP] in women. (**D**) Association between truncal fat mass and ln[hsCRP] in women. The dashed lines represent the fitted linear regression lines. The circle size indicates the frequency of observations. Pearson correlation coefficients (r) and corresponding *p*-values are shown in each panel.

Figure 1 shows the association between adiposity measurements and ln[hsCRP] according to sex. In men, both FMI and truncal fat mass were positively correlated with ln[hsCRP] (FMI: *r* = 0.232; truncal fat mass: *r* = 0.215; both *p* < 0.001). In women, stronger correlations were observed between adiposity and ln[hsCRP] levels (FMI, *r* = 0.373; truncal fat mass, *r* = 0.367; both *p* < 0.001). Increased adiposity was associated with higher ln[hsCRP] levels in both sexes, with stronger associations observed in women.

## 4. Discussion

In this nationally representative study, we used data from the KNHANES to investigate the association between detailed adiposity measures and systemic inflammation as assessed using hsCRP. Our findings indicate that both FMI and trunk fat mass were positively associated with ln[hsCRP]. Furthermore, the magnitude of these associations varied by sex and age, with stronger associations observed in women and younger individuals.

Our findings support the hypothesis that adipose tissue contributes to systemic inflammation. This is consistent with prior evidence that adipose tissue functions as an active endocrine organ that secretes pro-inflammatory cytokines [15]. In individuals with obesity, elevated levels of free fatty acids, ceramides, and diacylglycerol trigger pro-inflammatory serine kinase signaling pathways [16]. This molecular cascade promotes the release of cytokines, such as IL-6, which subsequently induces hepatic CRP synthesis [17,18]. The observed positive association between adiposity measures and ln[hsCRP] levels in this study is consistent with previous research demonstrating that increased body fat contributes to elevated systemic inflammatory activity. For example, relative body fat and waist circumference were strongly associated with hsCRP, fibrinogen, and white blood cell counts in a German population [19]. In addition, Liu et al. reported that the systemic immune-inflammation index, computed using lymphocyte, neutrophil, and platelet counts as its components, was associated with body fat distribution based on data from the National Health and Nutrition Examination Survey [20].

Our results highlight the advantages of body composition-based adiposity measures over BMI for evaluating obesity-related inflammation. BMI does not distinguish between lean mass and fat mass and may therefore underestimate the contribution of adiposity to metabolic risk. In contrast, indices such as FMI directly quantify fat mass relative to body size, providing a more accurate assessment of adiposity. The consistent association between FMI and hsCRP levels observed in our analysis suggests that FMI may serve as a more informative marker for examining the relationship between adiposity and inflammation.

In addition, central fat distribution, as assessed by truncal fat mass, was markedly associated with hsCRP levels. Abdominal fat accumulation, particularly visceral fat accumulation, is metabolically active and strongly associated with insulin resistance, dyslipidemia, and systemic inflammation [21]. In an Asian Indian population, visceral fat was found to be associated with insulin resistance, glucose intolerance, and elevated inflammatory markers, including CRP and TNF-α [22]. This may be driven by the overproduction of inflammatory mediators by immune cells, which are preferentially enriched in visceral fat in both lean and obese individuals [23]. The relationship between trunk fat mass and hsCRP levels supports the findings of previous studies, showing that central obesity is a strong determinant of cardiometabolic risk [24]. A potential mechanistic link between excess adiposity and systemic inflammation may involve the gut microbiota–adiposity–inflammation axis. Alterations in gut microbiota associated with increased adiposity may contribute to systemic inflammation. Consistent with this, recent clinical evidence suggests that microbiota modulation may be associated with inflammatory markers, including CRP-related outcomes [25], providing a possible biological explanation for these associations.

A notable finding of this study is the sexual dimorphism observed in the relationship between adiposity metrics and hsCRP levels. Specifically, both the correlation coefficients (r) and regression coefficients (β) were consistently higher in women than in men. These findings are consistent with those of previous studies reporting that the quantity and distribution of body fat influence CRP levels to a greater extent in women than in men [10,26]. In the present study, while waist circumference and BMI were higher in men than in women, PBF and FMI were markedly higher in women than in men. Trunk fat mass was comparable between the two sexes, whereas AFM was higher in women. Women typically have higher total body fat percentages and may exhibit distinct adipokine secretion profiles compared with males. Adiponectin was associated with hsCRP in both sexes, whereas leptin and plasminogen activator inhibitor-1 (PAI-1) were positively associated with hsCRP only in women [27]. Visceral adiposity was more strongly associated with adipokines (leptin and adipsin), inflammatory markers (CRP and PAI-1), and fibrosis-related biomarkers in women than in men [28]. Accordingly, women may be more susceptible to a heightened systemic inflammatory response to increased fat mass.

Hormonal factors, particularly estrogen-related effects on adipose tissue distribution and inflammatory pathways, may also influence these associations. Despite the anti-inflammatory and metabolically protective effects of estrogen, obesity-associated adiposity induces chronic inflammation and may override these benefits, partly through impaired estrogen receptor signaling and the development of estrogen resistance [29,30,31].

Age-specific analyses revealed that the association between adiposity and hsCRP levels was stronger in younger adults than in older adults. In the current study, the association between adiposity and inflammation was more pronounced in the younger group (aged 19–40 years) than in the older group (aged 41–70 years), which is consistent with previous findings demonstrating a stronger link between adiposity and inflammatory markers in younger populations [32,33]. One possible explanation is that age-related chronic low-grade inflammation and comorbid chronic conditions contribute to elevated inflammatory markers in older individuals, thereby attenuating the relative impact of adiposity alone [34]. In contrast, obesity appears to be the predominant driver of systemic inflammation in younger individuals. Furthermore, longitudinal evidence suggests that early-life adiposity is associated with elevated CRP levels in adulthood, underscoring the long-term impact of early metabolic dysregulation [35]. These findings suggest that early-life obesity represents a critical window for intervention, highlighting the importance of weight management in young adults for the prevention of future cardiovascular diseases.

This study has several strengths. First, it used data from a large, nationally representative population, which enhanced the generalizability of the findings. Second, detailed body composition measurements obtained through BIA enabled the assessment of specific adiposity parameters beyond traditional BMI. Third, we examined sex- and age-stratified associations, providing additional insights into the population subgroups in which adiposity-related inflammation may be particularly relevant.

Nevertheless, the limitations of this study must be addressed. The cross-sectional design of this study precluded causal inferences regarding the relationship between adiposity and systemic inflammation. In addition, although hsCRP is a widely used marker of inflammation, it may be influenced by acute infections, malignancies, autoimmune diseases, recent surgery, medication use, or other inflammatory conditions that were not fully captured in the dataset. Therefore, participants with these conditions could not be fully excluded, and residual confounding factors may have influenced the observed associations. Finally, body composition was measured using BIA rather than more precise imaging modalities such as dual-energy X-ray absorptiometry or computed tomography.

## 5. Conclusions

The present study demonstrate that FMI and truncal fat mass are independently associated with CRP levels in a large Korean population. These associations are more pronounced in women and younger individuals, highlighting sex- and age-related differences in the relationship between adiposity and inflammation. Collectively, these findings underscore the value of detailed body composition measures beyond BMI for assessing obesity-related inflammatory risk. In clinical practice, mildly elevated CRP levels without an apparent cause may be associated with obesity-related low-grade inflammation. From a public health perspective, these findings support the early identification of individuals with increased adiposity and inflammatory burden. Public health programs focusing on the early identification of high-risk individuals based on body fat distribution, along with targeted lifestyle interventions, may contribute to reducing metabolic risk in the Korean population.

## Figures and Tables

**Table 1:** Demographic and clinical characteristics of participants according to sex.

	Total (n = 32,635,626)	Male (n = 17,402,501)	Female (n = 15,233,125)
Age	49.6 ± 0.5	43.4 ± 0.6	51.0 ± 0.6
Current smoker	5,933,722 (18.2)	5,257,393 (30.2)	676,329 (4.4)
Alcohol consumption	17,830,478 (54.6)	11,659,734 (67.0)	6,170,743 (40.5)
Aerobic exercise	16,076,000 (49.3)	9,037,962 (51.9)	7,038,038 (46.2)
Waist circumference, cm	85.7 ± 0.3	90.0 ± 0.3	81.2 ± 0.3
BMI, kg/m^2^	24.7 ± 0.1	25.3 ± 0.1	24.0 ± 0.1
Hypertension	7,907,731 (24.2)	4,358,543 (25.1)	3,549,188 (23.3)
Diabetes	3,256,414 (10.0)	1,961,892 (11.3)	1,294,522 (8.5)
Dyslipidemia	7,183,621 (22.0)	3,321,295 (19.1)	3,862,326 (25.4)
SBP, mmHg	119.7 ± 0.4	122.5 ± 0.5	116.4 ± 0.5
DBP, mmHg	74.9 ± 0.3	77.1 ± 0.3	72.5 ± 0.3
Cholesterol, mg/dL	192.9 ± 1.0	191.2 ± 1.4	195.0 ± 1.1
HDL, mg/dL	55.7 ± 0.3	50.6 ± 0.4	61.5 ± 0.4
Triglyceride, mg/dL	138.6 ± 2.3	160.3 ± 3.8	113.7 ± 1.9
LDL, mg/dL	119.2 ± 0.8	119.5 ± 1.2	118.9 ± 1.0
HbA1c, %	5.59 ± 0.02	5.61 ± 0.02	5.57 ± 0.02
Glucose, mg/dL	101.3 ± 0.5	103.3 ± 0.7	98.9 ± 0.7
hsCRP, mg/L	1.38 ± 0.07	1.42 ± 0.1	1.34 ± 0.1
BFM, kg	20.12 ± 0.17	19.44 ± 0.21	20.89 ± 0.21
PBF, %	29.44 ± 0.17	25.42 ± 0.17	34.04 ± 0.19
FMI, kg/m^2^	7.4 ± 0.06	6.59 ± 0.07	8.34 ± 0.09
AFM, kg	8.58 ± 0.08	7.98 ± 0.09	9.26 ± 0.1
Fat mass of trunk, kg	10.4 ± 0.09	10.27 ± 0.11	10.54 ± 0.11

Values are presented as means ± standard errors or numbers (percentages). Alcohol consumption was defined as drinking alcohol ≥1 time per month. Aerobic exercise was defined as achieving a minimum of 150 min of moderate-intensity activity per week, 75 min of vigorous-intensity activity, or an equivalent combination. BMI, body mass index; SBP, systolic blood pressure; DBP, diastolic blood pressure; HbA1c, hemoglobin A1c (%); HDL, high-density lipoprotein cholesterol (mg/dL); LDL, low-density lipoprotein cholesterol (mg/dL); hsCRP, high-sensitivity C-reactive protein (mg/L); BFM, body fat mass; PBF, percentage body fat; FMI, fat mass index (kg/m^2^); AFM, appendicular fat mass (kg). FMI was calculated as BFM (kg) divided by height squared (m^2^). Appendicular fat mass was defined as the sum of the fat mass in both arms and legs (right arm, left arm, right leg, and left leg). Trunk fat mass (kg) was defined as fat mass measured in the trunk region.

**Table 2:** Pearson correlation coefficients between log-transformed hsCRP (ln[hsCRP]) and continuous variables in men and women.

Variables	Men		Women	
	Pearson Correlation	*p*-Value	Pearson Correlation	*p*-Value
Height, cm	−0.065	0.010	−0.025	0.283
Weight, kg	0.121	<0.001	0.318	<0.001
Waist circumference, cm	0.185	<0.001	0.319	<0.001
BMI, kg/m^2^	0.171	<0.001	0.348	<0.001
Systolic BP, mmHg	0.087	0.001	0.105	<0.001
Diastolic BP	0.037	0.137	0.077	0.001
HbA1c, %	0.111	<0.001	0.167	<0.001
Fasting glucose, mg/dL	0.083	0.001	0.170	<0.001
Total cholesterol, mg/dL	0.037	0.139	0.051	0.030
HDL cholesterol, mg/dL	−0.190	<0.001	−0.228	<0.001
Triglyceride, mg/dL	0.094	<0.001	0.198	<0.001
LDL cholesterol, mg/dL	0.046	0.069	0.081	0.001
BFM, kg	0.218	<0.001	0.374	<0.001
PBF, %	0.238	<0.001	0.330	<0.001
FMI, kg/m^2^	0.232	<0.001	0.373	<0.001
AFM, kg	0.219	<0.001	0.373	<0.001
Trunk fat mass, kg	0.215	<0.001	0.367	<0.001

BMI, body mass index; SBP, systolic blood pressure; DBP, diastolic blood pressure; HbA1c, hemoglobin A1c (%); HDL, high-density lipoprotein cholesterol (mg/dL); LDL, low-density lipoprotein cholesterol (mg/dL); hsCRP, high-sensitivity C-reactive protein (mg/L); BFM, body fat mass; PBF, percent body fat; FMI, fat mass index (kg/m^2^); AFM, appendicular fat mass (kg). FMI was calculated as BFM (kg) divided by height squared (m^2^). Appendicular fat mass was defined as the sum of the fat mass in both arms and legs (right arm, left arm, right leg, and left leg). Trunk fat mass (kg) was defined as fat mass measured in the trunk region.

**Table 3:** Association between fat mass index (FMI) and log-transformed high-sensitivity C-reactive protein (ln[hsCRP]): univariable and multivariable linear regression analyses stratified by sex.

Variable	Univariable Analysis		Multivariable Analysis Model 1		Multivariable Analysis Model 2	
	β (95% CI)	*p*-Value	β (95% CI)	*p*-Value	β (95% CI)	*p*-Value
Male						
Age	0 (0, 0.01)	0.077	0 (0, 0.01)	0.011	0.01 (0, 0.01)	0.002
Current smoker	0.09 (−0.02, 0.21)	0.116	0.13 (0.02, 0.25)	0.022	0.14 (0.02, 0.25)	0.022
Alcohol consumption	−0.09 (−0.2, 0.03)	0.145	−0.09 (−0.21, 0.02)	0.107	−0.09 (−0.21, 0.02)	0.115
Aerobic exercise	−0.05 (−0.16, 0.07)	0.429	0 (−0.11, 0.11)	0.994	0 (−0.11, 0.11)	0.956
Hypertension	0.16 (0, 0.33)	0.049			0 (−0.14, 0.14)	0.989
Dyslipidemia	0.07 (−0.06, 0.2)	0.271			−0.17 (−0.32, −0.02)	0.032
Diabetes	0.17 (0.05, 0.28)	0.004			−0.03 (−0.2, 0.14)	0.734
FMI	0.1 (0.07, 0.12)	<0.001	0.1 (0.08, 0.12)	<0.001	0.1 (0.08, 0.12)	<0.001
Female						
Age	0 (0, 0)	0.494	0 (−0.01, 0)	0.065	0 (0, 0)	0.769
Current smoker	0.09 (−0.12, 0.31)	0.398	−0.05 (−0.24, 0.14)	0.624	−0.04 (−0.23, 0.15)	0.695
Alcohol consumption	−0.03 (−0.14, 0.07)	0.515	0 (−0.11, 0.1)	0.951	−0.02 (−0.12, 0.09)	0.740
Aerobic exercise	−0.04 (−0.14, 0.06)	0.409	−0.03 (−0.13, 0.07)	0.582	−0.02 (−0.12, 0.07)	0.634
Hypertension	0.26 (0.06, 0.47)	0.012			−0.08 (−0.22, 0.05)	0.218
Dyslipidemia	−0.05 (−0.16, 0.06)	0.333			−0.19 (−0.31, −0.07)	0.002
Diabetes	0.01 (−0.1, 0.12)	0.866			0.08 (−0.12, 0.28)	0.417
FMI	0.14 (0.11, 0.16)	<0.001	0.14 (0.12, 0.16)	<0.001	0.14 (0.12, 0.16)	<0.001

Values are presented as β coefficients with 95% confidence intervals (CIs). Model 1 was adjusted for age, smoking status, alcohol consumption, and aerobic exercise status. Model 2 was adjusted for the variables included in Model 1, with additional adjustments for hypertension, dyslipidemia, and diabetes. Alcohol consumption was defined as drinking alcohol ≥1 time per month. Aerobic exercise was defined as achieving a minimum of 150 min of moderate-intensity activity per week, 75 min of vigorous-intensity activity, or an equivalent combination. FMI, fat mass index (kg/m^2^); BMI, body mass index (kg/m^2^).

**Table 4:** Association between trunk fat mass and log-transformed high-sensitivity C-reactive protein (ln[hsCRP]): univariable and multivariable linear regression analyses stratified by sex.

Variable	Univariable Analysis		Multivariable Analysis Model 1		Multivariable Analysis Model 2	
	β (95% CI)	*p*-Value	β (95% CI)	*p*-Value	β (95% CI)	*p*-Value
Male						
Age	0 (0, 0.01)	0.077	0.01 (0, 0.01)	0.0016	0.01 (0, 0.01)	0.001
Current smoker	0.09 (−0.02, 0.21)	0.116	0.13 (0.01, 0.24)	0.0314	0.13 (0.01, 0.24)	0.032
Alcohol consumption	−0.09 (−0.2, 0.03)	0.145	−0.11 (−0.23, 0)	0.0556	−0.11 (−0.23, 0)	0.058
Aerobic exercise	−0.05 (−0.16, 0.07)	0.429	0 (−0.11, 0.11)	0.9464	0 (−0.11, 0.11)	0.981
Hypertension	0.16 (0, 0.33)	0.049			0.01 (−0.13, 0.16)	0.837
Dyslipidemia	0.07 (−0.06, 0.2)	0.271			−0.16 (−0.31, −0.01)	0.033
Diabetes	0.17 (0.05, 0.28)	0.004			−0.02 (−0.19, 0.15)	0.799
Trunk fat mass	0.05 (0.04, 0.07)	<0.001	0.06 (0.04, 0.07)	<0.001	0.06 (0.05, 0.07)	<0.001
Female						
Age	0 (0, 0)	0.494	0 (0, 0)	0.413	0 (0, 0.01)	0.516
Current smoker	0.09 (−0.12, 0.31)	0.398	−0.04 (−0.23, 0.15)	0.697	−0.03 (−0.22, 0.16)	0.770
Alcohol consumption	−0.03 (−0.14, 0.07)	0.515	−0.01 (−0.12, 0.09)	0.786	−0.03 (−0.13, 0.08)	0.585
Aerobic exercise	−0.04 (−0.14, 0.06)	0.409	−0.03 (−0.13, 0.07)	0.513	−0.03 (−0.13, 0.07)	0.561
Hypertension	0.26 (0.06, 0.47)	0.012			−0.09 (−0.22, 0.05)	0.208
Dyslipidemia	−0.05 (−0.16, 0.06)	0.333			−0.19 (−0.31, −0.07)	0.002
Diabetes	0.01 (−0.1, 0.12)	0.866			0.08 (−0.12, 0.29)	0.416
Trunk fat mass	0.1 (0.09, 0.12)	<0.001	0.1 (0.08, 0.12)	<0.001	0.1 (0.09, 0.12)	<0.001

Values are presented as β coefficients with 95% confidence intervals (CIs). Model 1 was adjusted for age, smoking status, alcohol consumption, and aerobic exercise status. Model 2 was adjusted for variables included in Model 1, with additional adjustments for hypertension, dyslipidemia, and diabetes. Alcohol consumption was defined as drinking alcohol ≥1 time per month. Aerobic exercise was defined as achieving a minimum of 150 min of moderate-intensity activity per week, 75 min of vigorous-intensity activity, or an equivalent combination. BMI, body mass index (kg/m^2^).

**Table 5:** Sex- and age-stratified linear regression analyses of fat mass index and trunk fat mass in relation to log-transformed high-sensitivity C-reactive protein (ln[hsCRP]).

Variable		Univariable Analysis		Multivariable Analysis Model 1		Multivariable Analysis Model 2	
		β (95% CI)	*p*-Value	β (95% CI)	*p*-Value	β (95% CI)	*p*-Value
Male, total	Fat mass index	0.1 (0.07, 0.12)	<0.001	0.1 (0.08, 0.12)	<0.001	0.1 (0.08, 0.12)	<0.001
	Trunk fat mass, kg	0.05 (0.04, 0.07)	<0.001	0.06 (0.04, 0.07)	<0.001	0.06 (0.05, 0.07)	<0.001
Male aged 19–40 years	Fat mass index	0.13 (0.1, 0.16)	<0.001	0.13 (0.1, 0.16)	<0.001	0.13 (0.1, 0.16)	<0.001
	Trunk fat mass, kg	0.08 (0.06, 0.1)	<0.001	0.08 (0.06, 0.1)	<0.001	0.08 (0.06, 0.1)	<0.001
Male aged 41–70 years	Fat mass index	0.07 (0.04, 0.1)	<0.001	0.08 (0.05, 0.11)	<0.001	0.09 (0.06, 0.12)	<0.001
	Trunk fat mass, kg	0.04 (0.02, 0.06)	<0.001	0.04 (0.03, 0.06)	<0.001	0.05 (0.03, 0.07)	<0.001
Female, total	Fat mass index	0.14 (0.11, 0.16)	<0.001	0.14 (0.12, 0.16)	<0.001	0.14 (0.12, 0.16)	<0.001
	Trunk fat mass, kg	0.1 (0.09, 0.12)	<0.001	0.1 (0.08, 0.12)	<0.001	0.1 (0.09, 0.12)	<0.001
Female aged 19–40 years	Fat mass index	0.18 (0.14, 0.21)	<0.001	0.18 (0.15, 0.21)	<0.001	0.18 (0.15, 0.21)	<0.001
	Trunk fat mass	0.14 (0.11, 0.16)	<0.001	0.14 (0.11, 0.16)	<0.001	0.14 (0.11, 0.17)	<0.001
Female aged 41–70 years	Fat mass index	0.13 (0.1, 0.16)	<0.001	0.13 (0.1, 0.16)	<0.001	0.14 (0.11, 0.16)	<0.001
	Trunk fat mass	0.09 (0.07, 0.11)	<0.001	0.09 (0.07, 0.11)	<0.001	0.1 (0.07, 0.12)	<0.001

Model 1 was adjusted for age, smoking status, alcohol consumption, and aerobic exercise status. Model 2 was adjusted for the variables included in Model 1, with additional adjustments for hypertension, dyslipidemia, and diabetes.

## Data Availability

The data presented in this study are publicly available from the Korea National Health and Nutrition Examination Survey database (https://knhanes.kdca.go.kr, accessed on 11 August 2024).

## Supplementary Materials

The following supporting information can be downloaded at: https://www.mdpi.com/article/10.3390/medicina62061014/s1. Table S1: Interaction analyses for the associations of fat mass index and trunk fat mass with log-transformed high-sensitivity C-reactive protein (ln[hsCRP]) according to sex and age group.

## Abbreviations

The following abbreviations are used in this manuscript:

AFM	Appendicular fat mass
BFM	Body fat mass
BIA	Bioelectrical impedance analysis
BMI	Body mass index
CI	Confidence intervals
CRP	C-reactive protein
DBP	Diastolic blood pressure
FMI	Fat mass index
HbA1c	Hemoglobin A1c
HDL	High-density lipoprotein cholesterol
KNHANES	Korea National Health and Nutrition Examination Survey
LDL	Low-density lipoprotein cholesterol
PBF	Percentage body fat
SBP	Systolic blood pressure
