# Supplementary material for: The Association Between Serum C-Reactive Protein Levels and Body Fat Parameters: Results from the Korean National Health and Nutrition Examination Survey
Source: Medicina (Kaunas). 2026 May 23;62(6):1014. doi: 10.3390/medicina62061014 (PMC13304235; doi:10.3390/medicina62061014)
Supplement: Supplementary file 1 [file medicina-62-01014-s001.zip › medicina-4290049-supplementary.pdf]

**Supplementary Table S1.** Interaction analyses for the associations of fat mass index and trunk fat mass with log-transformed high-sensitivity C-reactive protein (ln[hsCRP]) according to sex and age group.

| Variable | β (95% CI) | p-value |
|---|---|---|
| FMI | 0.13 (0.11–0.16) | <0.001 |
| FMI × female sex | 0.05 (0.02–0.07) | 0.001 |
| FMI × age 41–70 years | −0.05 (−0.08 to −0.02) | 0.004 |
| Truncal fat mass | 0.08 (0.06–0.10) | <0.001 |
| Truncal fat mass × female sex | 0.05 (0.03–0.07) | <0.001 |
| Truncal fat mass × age 41–70 years | −0.04 (−0.06 to −0.02) | <0.001 |

Values are presented as β coefficients with 95% confidence intervals (CIs).
Models were adjusted for age, smoking status, alcohol consumption, aerobic exercise, hypertension, dyslipidemia, and diabetes.
FMI, fat mass index (kg/m$^2$).
